# Sodium-Glucose Cotransporter 2 (SGLT2) Inhibitors in Non-diabetic Conditions Beyond Heart Failure and Chronic Kidney Disease: Emerging Evidence, Mechanisms, and Practical Considerations

**DOI:** 10.7759/cureus.104727

**Published:** 2026-03-05

**Authors:** Digantkumar Patel, Tejas Patel

**Affiliations:** 1 Hospital Medicine, Springfield Clinic, Springfield, USA

**Keywords:** gout, hypertension, hyperuricemia, metabolic dysfunction-associated steatotic liver disease, nonalcoholic fatty liver disease, non-diabetic populations, obesity, sglt2 inhibitors, sodium-glucose cotransporter 2 inhibitors, syndrome of inappropriate antidiuretic hormone secretion

## Abstract

Sodium-glucose cotransporter 2 (SGLT2) inhibitors were developed as glucose-lowering agents, yet their clinical impact has expanded well beyond glycemic control. Although their benefits in heart failure and chronic kidney disease are now well established, a growing body of mechanistic and clinical evidence suggests broader utility in non-diabetic conditions such as nonalcoholic fatty liver disease (NAFLD), obesity and visceral adiposity, hypertension, hyperuricemia/gout, atrial fibrillation risk modulation, and select disorders of water balance (e.g., syndrome of inappropriate antidiuresis). These effects appear to arise from convergent pathways including natriuresis and osmotic diuresis, improved tubuloglomerular signaling, hemodynamic unloading, reductions in visceral fat and inflammation, alterations in urate handling, and shifts in substrate utilization with potential mitochondrial and endothelial benefits. This narrative review synthesizes emerging evidence for SGLT2 inhibitors in non-diabetic indications beyond heart failure and chronic kidney disease, with emphasis on biologic rationale, outcomes data, patient selection, safety in normoglycemic populations, and future research priorities.

## Introduction and background

Sodium-glucose cotransporter 2 (SGLT2) inhibitors were initially developed as antihyperglycemic agents for the management of type 2 diabetes mellitus by promoting renal glucose excretion through inhibition of proximal tubular glucose reabsorption. Early clinical adoption focused primarily on glycemic control and modest weight reduction. However, over the past decade, large cardiovascular and renal outcome trials have fundamentally reshaped the understanding of this drug class, revealing benefits that extend far beyond glucose lowering. These discoveries have positioned SGLT2 inhibitors as cornerstone therapies in the management of heart failure and chronic kidney disease, irrespective of diabetic status. As clinical experience has expanded, it has become increasingly evident that the physiologic effects of SGLT2 inhibition intersect with multiple organ systems and disease pathways, raising important questions about their potential role in non-diabetic conditions beyond heart failure and chronic kidney disease.

The broad therapeutic profile of SGLT2 inhibitors reflects their unique mechanism of action at the level of the renal proximal tubule, where glucose transport is tightly coupled to sodium handling. Inhibition of SGLT2 leads not only to glucosuria but also to natriuresis, osmotic diuresis, and alterations in intrarenal hemodynamics. These renal effects translate into downstream systemic consequences, including reductions in blood pressure, plasma volume, and arterial stiffness, as well as favorable changes in cardiac loading conditions. Importantly, many of these effects occur independently of baseline glycemic status, providing a physiologic rationale for benefit in normoglycemic individuals. As a result, SGLT2 inhibitors are increasingly conceptualized not simply as glucose-lowering agents, but as cardio-renal-metabolic modulators.

The recognition that SGLT2 inhibitors exert pleiotropic effects has prompted growing interest in their application across a range of non-diabetic disease states characterized by shared pathophysiologic features such as obesity, visceral adiposity, insulin resistance, inflammation, endothelial dysfunction, neurohormonal activation, and abnormal fluid handling. These conditions frequently coexist in clinical practice, particularly among patients with metabolic syndrome, fatty liver disease, hypertension, and disorders of water balance. From a population health perspective, the convergence of these conditions represents a substantial and growing burden of morbidity, underscoring the importance of therapeutic strategies that can address multiple pathways simultaneously.

Nonalcoholic fatty liver disease (NAFLD), recently reframed as metabolic dysfunction-associated steatotic liver disease (MASLD), exemplifies this intersection of metabolic, inflammatory, and vascular dysfunction. NAFLD affects a large proportion of adults worldwide and is strongly associated with obesity, insulin resistance, hypertension, and dyslipidemia, even in the absence of overt diabetes. Current therapeutic options remain limited, with lifestyle modification as the primary intervention and no widely approved pharmacologic therapy for most patients. The observation that SGLT2 inhibitors reduce visceral adiposity, improve metabolic efficiency, and lower systemic inflammation has generated interest in their potential to modify hepatic steatosis and liver injury. Importantly, emerging data suggest that reductions in liver fat may occur even in individuals without diabetes, highlighting a possible role for SGLT2 inhibitors in a population with substantial unmet clinical need.

Obesity and overweight states represent another area of growing interest. Although SGLT2 inhibitors do not produce weight loss of the magnitude seen with dedicated anti-obesity pharmacotherapies, they consistently result in modest reductions in body weight and waist circumference through sustained caloric loss and metabolic shifts. In non-diabetic individuals with obesity-related risk factors, even modest weight reduction can translate into meaningful improvements in blood pressure, lipid profiles, hepatic steatosis, and overall cardiometabolic risk. This raises the possibility that SGLT2 inhibitors may serve as adjunctive therapies within a broader risk-reduction framework rather than as primary weight-loss agents.

Hypertension remains one of the most prevalent and impactful cardiovascular risk factors globally, and its pathogenesis often involves abnormalities in sodium handling, vascular tone, and neurohormonal activation. The natriuretic and hemodynamic effects of SGLT2 inhibitors result in modest but sustained reductions in blood pressure across diverse patient populations. Notably, these effects appear largely independent of glucose-lowering and are observed even in normoglycemic individuals. While SGLT2 inhibitors are unlikely to replace standard antihypertensive therapies, their ability to provide incremental blood pressure reduction may be particularly valuable in patients with resistant hypertension or in those with coexisting metabolic and renal risk factors.

Beyond metabolic and hemodynamic effects, SGLT2 inhibitors influence uric acid metabolism by increasing renal urate excretion. Hyperuricemia is increasingly recognized not only as the biochemical substrate for gout but also as a contributor to systemic inflammation, endothelial dysfunction, and cardiovascular risk. Observational and trial-based evidence has demonstrated consistent reductions in serum uric acid levels among individuals treated with SGLT2 inhibitors, along with signals suggesting reduced incidence of gout events. These findings have generated interest in the potential role of SGLT2 inhibitors in patients with hyperuricemia or recurrent gout, including those without diabetes, although definitive outcome data in normoglycemic populations remain limited.

Disorders of water balance represent a more novel and mechanistically distinct potential application of SGLT2 inhibitors. The syndrome of inappropriate antidiuresis (SIAD) is characterized by impaired free-water excretion, leading to chronic hyponatremia with associated cognitive, gait, and fracture risks. Traditional therapies for SIAD are often limited by tolerability, cost, or variable efficacy. By promoting osmotic diuresis and increasing electrolyte-free water clearance, SGLT2 inhibitors have emerged as a potential adjunctive therapy for chronic SIAD. Early randomized and observational data suggest that empagliflozin may modestly increase serum sodium concentrations in selected patients, offering a new physiologic approach to a challenging clinical problem.

Emerging evidence has also linked SGLT2 inhibitors to reductions in atrial fibrillation incidence and improvements in arrhythmia-related outcomes, primarily derived from secondary analyses of large cardiovascular outcome trials. Although these findings have largely been observed in populations with diabetes, the proposed mechanisms, such as reductions in atrial stretch, inflammation, oxidative stress, and autonomic imbalance, are not inherently diabetes-specific. As atrial fibrillation frequently coexists with obesity, hypertension, and metabolic dysfunction, understanding whether these benefits extend to non-diabetic populations is an important area of ongoing investigation.

Similarly, obstructive sleep apnea (OSA) has been proposed as a potential target for SGLT2 inhibitor therapy based on indirect mechanisms, including reductions in visceral fat, fluid redistribution, and cardiometabolic inflammation. While current evidence remains preliminary and heterogeneous, the hypothesis reflects a broader recognition that SGLT2 inhibitors may influence multisystem disease processes beyond traditional cardiometabolic endpoints.

Polycystic ovary syndrome (PCOS) represents another complex condition characterized by metabolic dysfunction, insulin resistance, and increased long-term cardiovascular risk, often in the absence of overt diabetes. Early studies evaluating SGLT2 inhibitors in PCOS populations have focused on metabolic and anthropometric outcomes, with some signals of benefit. However, their role in addressing reproductive and hyperandrogenic manifestations remains uncertain, and further investigation is needed before routine clinical use can be considered.

Despite the expanding interest in non-diabetic applications of SGLT2 inhibitors, several important challenges remain. Most existing evidence is derived from studies that include or are enriched for patients with diabetes, raising questions about generalizability to normoglycemic populations. Additionally, the balance of benefits and risks may differ in non-diabetic individuals, particularly with respect to volume status, genital infections, and rare but serious adverse events such as euglycemic ketoacidosis. Careful patient selection, monitoring, and clinical judgment are therefore essential when considering off-label or investigational use.

Given these considerations, a comprehensive synthesis of the available evidence is needed to clarify where SGLT2 inhibitors show the greatest promise beyond heart failure and chronic kidney disease, where evidence remains preliminary, and how these agents might be responsibly integrated into clinical practice or future research. This narrative review aims to critically evaluate the mechanistic rationale, clinical data, safety considerations, and evidence gaps related to the use of SGLT2 inhibitors in non-diabetic conditions beyond heart failure and chronic kidney disease, with the goal of informing clinicians, researchers, and policymakers as this therapeutic landscape continues to evolve.

This article is intended as a narrative review rather than a systematic review. The objective was to synthesize emerging mechanistic and clinical evidence regarding potential non-diabetic applications of SGLT2 inhibitors beyond heart failure and chronic kidney disease. Relevant literature, including randomized trials, meta-analyses, observational studies, and mechanistic reviews, was identified through targeted searches of major biomedical databases and manual review of references. Given the exploratory and emerging nature of this topic, a formal systematic review methodology or Preferred Reporting Items for Systematic Reviews and Meta-Analyses (PRISMA) framework was not applied.

## Review

Mechanistic basis for “beyond-glucose” effects

SGLT2 inhibitors exert their primary pharmacologic action by blocking glucose-sodium co-transport in the proximal renal tubule, leading to glucosuria accompanied by mild natriuresis and osmotic diuresis. These renal effects extend beyond glycemic lowering and generate systemic physiologic consequences that are relevant even in individuals without diabetes. By reducing proximal tubular sodium reabsorption, SGLT2 inhibitors restore tubuloglomerular feedback and alter downstream hemodynamic and neurohormonal signaling. Mechanistic syntheses have emphasized that these renal actions translate into reductions in plasma and interstitial volume, improvements in vascular function, and modulation of sympathetic tone, all of which may contribute to cardiovascular and metabolic benefits independent of glucose control [1-5].

Beyond hemodynamics, SGLT2 inhibitors influence adiposity, inflammation, and substrate utilization. Clinical and translational studies have demonstrated modest but consistent reductions in body weight, particularly visceral adiposity, along with favorable changes in inflammatory markers and adipokines. These effects may reflect a combination of caloric loss through glucosuria, shifts toward lipid oxidation, and improvements in insulin sensitivity surrogates [6-9]. In parallel, SGLT2 inhibitors enhance urate excretion by altering tubular handling of uric acid, resulting in lower serum urate levels that may reduce gout risk and attenuate urate-associated vascular inflammation [10-12]. Hepatic effects have also been observed, including reductions in liver fat content and transaminase levels, potentially mediated by decreased adipose-liver flux, reduced de novo lipogenesis, and anti-inflammatory signaling [13-16]. Finally, osmotic diuresis and altered tubular physiology may increase free-water clearance, providing a biologic rationale for investigation in disorders of water balance such as chronic SIAD [17-19]. Collectively, these pleiotropic mechanisms provide a coherent biologic foundation for exploring SGLT2 inhibitors across a range of non-diabetic conditions beyond heart failure and chronic kidney disease.

The pleiotropic effects of SGLT2 inhibitors extend beyond glycemic modulation and involve interconnected renal, metabolic, and vascular pathways. Table 1 summarizes key mechanistic domains linking SGLT2 inhibition to observed benefits in non-diabetic conditions [1-6,10-19].

**Table 1:** Pathophysiologic mechanisms linking SGLT2 inhibition to non-diabetic clinical benefits beyond heart failure and CKD AF: atrial fibrillation; BP: blood pressure; NAFLD: nonalcoholic fatty liver disease; OSA: obstructive sleep apnea; SIAD: syndrome of inappropriate antidiuresis; SGLT2: sodium-glucose cotransporter 2; CKD: chronic kidney disease; MASLD: metabolic dysfunction-associated steatotic liver disease

Mechanistic pathway	Primary renal or systemic effect	Downstream clinical implications	Relevant non-diabetic conditions
Proximal tubular natriuresis	Increased sodium delivery to macula densa; restoration of tubuloglomerular feedback	Reduced intraglomerular pressure; modest blood pressure reduction	Hypertension, vascular risk states
Osmotic diuresis	Increased free-water clearance with mild volume contraction	Improved congestion; correction of dilutional hyponatremia	Chronic SIAD
Reduced visceral adiposity	Caloric loss via glucosuria; altered fat distribution	Decreased hepatic fat, inflammatory cytokines	NAFLD/MASLD, obesity
Improved endothelial function	Reduced oxidative stress and arterial stiffness	Improved vascular compliance; BP lowering	Hypertension, AF risk modulation
Altered substrate utilization	Shift toward lipid oxidation and ketone availability	Improved metabolic efficiency, mitochondrial function	NAFLD, obesity-related risk
Enhanced uricosuria	Reduced proximal tubular urate reabsorption	Lower serum uric acid; reduced gout risk	Hyperuricemia, gout
Anti-inflammatory signaling	Reduced adipokines and systemic inflammatory markers	Potential reduction in arrhythmogenic substrate	AF risk, metabolic syndrome
Modulation of sympathetic tone	Reduced plasma volume and arterial stiffness	Lower heart rate variability stress	Hypertension, OSA-related risk

NAFLD and MASLD

NAFLD, now increasingly framed as MASLD, is closely linked to visceral adiposity, systemic inflammation, and cardiometabolic risk. Even in the absence of overt diabetes, interventions that reduce visceral fat and improve metabolic milieu may favorably influence hepatic steatosis and liver injury. SGLT2 inhibitors, through their effects on adiposity, inflammation, and substrate utilization, therefore represent a biologically plausible therapeutic approach in this population. Clinical evidence supporting this hypothesis has grown in recent years. A randomized trial demonstrated that empagliflozin reduced liver fat content in individuals both with and without type 2 diabetes, suggesting that hepatic benefits may not be strictly diabetes-dependent [13]. A 2024 systematic review and meta-analysis further reported improvements in imaging-based steatosis measures and select fibrosis-related indices among patients with NAFLD treated with SGLT2 inhibitors, although many included studies were enriched for diabetic populations [14]. Additional contemporary meta-analyses and narrative syntheses have consistently described reductions in alanine aminotransferase levels and improvements in non-invasive steatosis markers, typically accompanied by modest weight loss and cardiometabolic improvements [15,16]. From a practical standpoint, the most consistent signal in non-diabetic NAFLD relates to reductions in liver fat and transaminase levels, while evidence for histologic endpoints such as steatohepatitis resolution or fibrosis regression remains insufficient in normoglycemic cohorts. Patients with NAFLD who also exhibit obesity or metabolic syndrome features may represent the most rational population for future trials and careful clinical consideration, given the potential for multi-domain metabolic benefits.

Obesity and overweight in non-diabetic populations

SGLT2 inhibition induces caloric loss through glucosuria and may shift energy balance toward increased lipid utilization. Although compensatory increases in appetite may attenuate weight loss, clinical studies have consistently reported modest reductions in body weight and waist circumference across diverse populations, including individuals without diabetes. These effects have generated interest in the potential role of SGLT2 inhibitors as adjunctive agents in obesity management. Evidence in non-diabetic obesity remains limited but informative. In one randomized study of obese adults without diabetes, dapagliflozin combined with once-weekly exenatide produced sustained reductions in body weight and systolic blood pressure over 52 weeks and reduced the prevalence of prediabetes, highlighting a potential role for combination strategies targeting complementary pathways [7]. Systematic reviews comparing pharmacologic approaches to weight reduction have consistently noted that SGLT2 inhibitors produce modest weight loss relative to incretin-based anti-obesity therapies, yet these reductions may still be clinically meaningful when integrated into broader cardiometabolic risk-reduction strategies [8,9]. Clinically, expectations should be calibrated toward modest rather than transformative weight reduction, typically on the order of a few kilograms. SGLT2 inhibitors may therefore find niche roles in patients with obesity who also have hypertension, NAFLD, hyperuricemia, or other clustered cardiometabolic risk factors, where incremental benefits across multiple domains are desirable.

Hypertension and vascular risk modulation

The natriuretic and osmotic diuretic effects of SGLT2 inhibitors provide a mechanistic basis for blood pressure reduction that appears partially independent of glycemic status. By reducing intravascular volume, improving arterial compliance, and modulating neurohormonal pathways, these agents may exert modest antihypertensive effects across a range of populations. A 2020 American Heart Association review detailed the renal and vascular mechanisms underlying the blood pressure-lowering effects of SGLT2 inhibitors, emphasizing natriuresis, reduced intraglomerular pressure, and improved endothelial function [4]. Meta-analyses have consistently demonstrated modest but clinically meaningful reductions in systolic blood pressure, often in the range of approximately 3-5 mmHg, across heterogeneous study populations [6]. More recent analyses synthesizing randomized trial data have suggested that blood pressure lowering is not tightly linked to baseline estimated glomerular filtration rate, supporting broader physiologic generalizability beyond diabetic or chronic kidney disease populations [19]. In practice, the magnitude of blood pressure reduction associated with SGLT2 inhibitors is comparable to adding a mild antihypertensive agent and is unlikely to replace guideline-directed therapy. Nonetheless, these effects may provide an additive benefit, particularly in patients with coexisting metabolic risk factors. Careful attention to volume status, orthostatic symptoms, and concomitant diuretic therapy is essential, especially in older adults.

Hyperuricemia and gout risk reduction

Uric acid metabolism represents a distinctive mechanistic bridge between renal tubular physiology and systemic inflammatory risk. SGLT2 inhibitors consistently increase uricosuria, resulting in lower serum urate levels, a property that has attracted interest for hyperuricemia and gout risk reduction. A 2025 meta-analysis demonstrated that SGLT2 inhibitors lowered serum uric acid levels compared with placebo in populations with and without diabetes, reinforcing a class effect on urate handling [11]. A 2024 network meta-analysis of randomized cardiovascular outcome trials further reported reduced gout events among patients treated with SGLT2 inhibitors compared with placebo and some active comparators, although most data were derived from diabetes-enriched populations [12]. Complementing these findings, a 2024 Journal of the American College of Cardiology review synthesized mechanistic and clinical evidence linking SGLT2 inhibition to improved urate metabolism, reduced hyperuricemia, and potential downstream vascular benefits [10]. Clinically, the most robust evidence supports a consistent urate-lowering effect rather than definitive gout prevention in non-diabetic populations. Patients with metabolic syndrome features and recurrent gout risk may represent an attractive population for future dedicated trials, while real-world use requires careful balancing of potential benefits and safety considerations.

Atrial fibrillation, stroke, and broader cardiovascular endpoints

Although heart failure and chronic kidney disease are excluded from the primary scope of this review, arrhythmia and vascular outcomes have been extensively evaluated in large cardiovascular outcome trials and meta-analyses. Emerging signals suggest that SGLT2 inhibitors may influence atrial fibrillation and stroke risk through mechanisms that extend beyond glucose lowering. A 2021 meta-analysis published in Stroke reported a reduction in atrial fibrillation incidence and a possible reduction in hemorrhagic stroke among patients treated with SGLT2 inhibitors, findings largely derived from diabetes cardiovascular outcome trials but supportive of mechanistic hypotheses involving hemodynamic unloading and reduced inflammation [20-22]. A subsequent 2023 meta-analysis similarly reported a lower incidence of atrial fibrillation across included studies [23]. At present, these findings should be interpreted as hypothesis-supporting rather than definitive for non-diabetic primary prevention. The most defensible clinical posture is to view potential arrhythmia and stroke benefits as ancillary to broader cardiometabolic optimization rather than as stand-alone indications.

Obstructive sleep apnea (OSA)

OSA is closely linked to obesity, visceral fat accumulation, fluid redistribution, and systemic inflammation-pathophysiologic domains that overlap with the known effects of SGLT2 inhibitors. These shared mechanisms have prompted interest in whether SGLT2 inhibition may modulate OSA severity or risk. A 2023 review proposed SGLT2 inhibitors as candidates for OSA risk modification, emphasizing mechanistic plausibility and the need for dedicated clinical trials [24]. A 2025 evidence synthesis reported improvements in apnea-hypopnea indices and oxygenation metrics in available studies, although most data were derived from diabetes-enriched populations and exhibited substantial heterogeneity [25]. Current evidence remains preliminary, and OSA should not be considered a direct therapeutic target for SGLT2 inhibitors. However, patients with OSA who also have obesity, NAFLD, or hypertension may represent rational populations for future mechanistic and outcomes-focused studies.

Polycystic ovary syndrome (PCOS)

PCOS is characterized by insulin resistance, adiposity, and increased cardiometabolic risk, even in the absence of overt diabetes. These features provide a biologic rationale for exploring SGLT2 inhibitors as metabolic modulators in this population. A 2022 meta-analysis reported that SGLT2 inhibitors improved metabolic and anthropometric parameters in women with PCOS and suggested potential favorable effects on hormonal profiles in some analyses [26]. However, the available evidence is largely derived from small studies with short follow-up durations. At present, SGLT2 inhibitors remain non-standard therapy for PCOS. Future trials should prioritize patient-centered outcomes, including reproductive endpoints, hyperandrogenism symptoms, and long-term cardiometabolic risk reduction.

Chronic SIAD

Chronic SIAD represents a novel and physiologically driven application for SGLT2 inhibitors. Through osmotic diuresis and altered tubular handling of solute and water, empagliflozin may enhance free-water clearance and increase serum sodium levels without direct vasopressin antagonism. A randomized trial published in 2020 demonstrated that empagliflozin increased plasma sodium concentrations in patients with SIAD, with acceptable tolerability [17]. A subsequent 2024 clinical commentary and review discussed emerging outpatient experience and emphasized cautious implementation, close sodium monitoring, and the need for larger confirmatory studies [18]. Among non-diabetic, non-heart failure indications, SIAD represents one of the most targeted applications of SGLT2 inhibitors with randomized evidence. Careful patient selection and monitoring are essential to avoid overly rapid correction of hyponatremia.

Safety considerations in non-diabetic populations

While large cardiovascular outcome trials have established broad safety profiles for SGLT2 inhibitors in diabetes, their use in non-diabetic populations necessitates careful risk-benefit assessment. Genital mycotic infections remain a class effect and require patient education regarding hygiene, early symptom recognition, and prompt treatment [27-29]. Volume depletion and orthostatic hypotension may occur, particularly in older adults, those with low baseline blood pressure, or patients receiving concomitant diuretics, underscoring the importance of individualized dosing and medication review [4,6,20]. Euglycemic ketoacidosis, although rare, represents a serious adverse event, with risk increased during prolonged fasting, acute illness, perioperative states, very low-carbohydrate diets, and heavy alcohol use. Temporary drug discontinuation during high-risk periods is recommended [30-32]. Urinary tract infection risk appears modest overall but warrants individualized assessment, particularly in patients with recurrent infections [27-29]. Historical signals regarding bone fractures and amputations, primarily associated with canagliflozin in select datasets, should be interpreted with nuance and considered in patients with severe peripheral arterial disease or prior amputations [33].

Given that many proposed applications involve normoglycemic individuals, safety considerations warrant particular attention. Table 2 summarizes adverse effects and mitigation strategies relevant to non-diabetic populations receiving SGLT2 inhibitors [27-33].

**Table 2:** Safety issues relevant to non-diabetic use BP: blood pressure; PAD: peripheral arterial disease; UTI: urinary tract infection

Safety issue	Why it matters in non-diabetics	Practical mitigation
Volume depletion/orthostasis	BP lowering + diuretic effect may be less “buffered” by hyperglycemia	Review diuretics, counsel hydration, monitor BP, and symptoms
Genital mycotic infections	Class effect independent of diabetes	Education, early treatment plans
Euglycemic ketoacidosis (rare)	Can occur with low intake/fasting/illness, even without diabetes	Hold for acute illness/fasting; perioperative holds; avoid extreme low-carb during illness
UTIs	Usually modest risk, but relevant to recurrent UTI patients	Individualize, counsel, monitor
PAD/amputation concern (agent-specific history)	Mostly historical signal; still relevant to high-risk limb ischemia	Avoid/individualize in severe PAD; foot care vigilance

Strength of evidence and investigational domains

Among non-heart failure, non-chronic kidney disease indications, the most mature evidence supports reductions in liver fat and transaminase levels in NAFLD, modest blood pressure lowering across populations, consistent serum urate reduction with supportive gout-risk signals, and improvement in serum sodium levels in chronic SIAD with randomized evidence [4,6,10-21]. In contrast, applications such as OSA, PCOS, and atrial fibrillation or stroke prevention in non-diabetic populations remain investigational, with current data primarily hypothesis-generating [22-26]. Future research should prioritize well-designed randomized trials in normoglycemic populations, focusing on patient-centered outcomes, safety, and long-term clinical benefit to define the appropriate role of SGLT2 inhibitors beyond their established indications.

To contextualize the heterogeneity of evidence supporting non-diabetic uses of SGLT2 inhibitors beyond heart failure and chronic kidney disease, Table 3 summarizes the strength of available data, observed clinical effects, and current clinical readiness across emerging indications.

**Table 3:** Non-diabetic clinical applications of SGLT2 inhibitors beyond heart failure and chronic kidney disease: evidence strength, clinical effects, and readiness * Clinical readiness refers to cautious off-label consideration or research applicability in selected patients and does not imply guideline endorsement. AF: atrial fibrillation; ALT: alanine aminotransferase; BP: blood pressure; CVOT: cardiovascular outcomes trial; MASLD: metabolic dysfunction-associated steatotic liver disease; NAFLD: nonalcoholic fatty liver disease; OSA: obstructive sleep apnea; PCOS: polycystic ovary syndrome; RCT: randomized controlled trial; SIAD: syndrome of inappropriate antidiuresis; SGLT2: sodium-glucose cotransporter 2

Non-diabetic condition	Most consistent observed effects	Type of supporting evidence	Evidence consistency	Clinical readiness*	Key limitations
NAFLD/MASLD [13-16]	Reduced hepatic fat content; improvement in ALT and metabolic indices	RCTs, meta-analyses, imaging-based studies	Moderate	Emerging	Limited histologic outcomes; many studies include diabetic cohorts
Obesity/overweight [7-9]	Modest weight loss; reduction in visceral adiposity; BP lowering	RCTs, metabolic and mechanistic studies	Low-moderate	Adjunctive	Weight loss modest; compensatory caloric intake
Hypertension [4,6,20]	Modest sustained reduction in systolic and diastolic BP	Meta-analyses, physiologic trials	Moderate	Adjunctive	BP effect is insufficient as monotherapy
Hyperuricemia/gout [10-12,21]	Lower serum uric acid; reduced gout event risk signal	Meta-analyses; CVOT secondary analyses	Moderate	Promising	Gout outcomes are largely derived from diabetes trials
Chronic SIAD [17-19]	Increase in serum sodium via enhanced free-water clearance	Randomized trial; clinical series	Moderate	Selective use	Limited long-term outpatient safety data
Atrial fibrillation risk [22,23]	Reduced the incident AF signal	Meta-analyses of cardiovascular outcome trials	Low-moderate	Investigational	Indirect evidence; no AF-targeted trials
OSA [24,25]	Possible reduction in apnea-hypopnea index and symptom burden	Reviews; observational and post-hoc analyses	Low	Experimental	Heterogeneous populations; few dedicated trials
PCOS [26]	Improvement in metabolic and anthropometric parameters	Small trials; meta-analyses	Low	Experimental	Limited fertility and hyperandrogenism outcomes

## Conclusions

SGLT2 inhibitors are increasingly understood as cardio-metabolic-renal modulators rather than purely glucose-lowering agents. Beyond heart failure and chronic kidney disease, the most compelling emerging non-diabetic applications include NAFLD (especially liver fat reduction), modest but consistent blood pressure lowering, urate reduction with plausible gout-risk mitigation, and targeted use in chronic SIAD supported by randomized evidence. Other domains, OSA, PCOS, and arrhythmia/vascular prevention, remain promising but investigational, with current signals largely indirect or derived from diabetes-enriched datasets. Until dedicated non-diabetic outcome trials mature, clinicians should treat these indications as evidence-informed opportunities for research and careful individualized decision-making, balancing multi-domain benefits against predictable class adverse effects and rare serious complications.
